# Leptin Receptors Are Not Required for Roux-en-Y Gastric Bypass Surgery to Normalize Energy and Glucose Homeostasis in Rats

**DOI:** 10.3390/nu13051544

**Published:** 2021-05-04

**Authors:** Mohammed K. Hankir, Laura Rotzinger, Arno Nordbeck, Caroline Corteville, Ulrich Dischinger, Juna-Lisa Knop, Annett Hoffmann, Christoph Otto, Florian Seyfried

**Affiliations:** 1 Department of General, Visceral, Vascular and Pediatric Surgery, University Hospital Wuerzburg, 97080 Wuerzburg, Germany; laura.rotzinger@googlemail.com (L.R.); arno.nordbeck@gmx.de (A.N.); corteville_c@ukw.de (C.C.); knop_j@ukw.de (J.-L.K.); hoffmann_a4@ukw.de (A.H.); otto_c@ukw.de (C.O.); 2 Department of Endocrinology, University Hospital Wuerzburg, 97080 Wuerzburg, Germany; dischinger_u@ukw.de

**Keywords:** Roux-en-Y gastric bypass surgery, energy homeostasis, glucose homeostasis, fatty liver, leptin system, Zucker fatty *fa/fa* rats

## Abstract

Sensitization to the adipokine leptin is a promising therapeutic strategy against obesity and its comorbidities and has been proposed to contribute to the lasting metabolic benefits of Roux-en-Y gastric bypass (RYGB) surgery. We formally tested this idea using Zucker fatty *fa/fa* rats as an established genetic model of obesity, glucose intolerance, and fatty liver due to leptin receptor deficiency. We show that the changes in body weight in these rats following RYGB largely overlaps with that of diet-induced obese Wistar rats with intact leptin receptors. Further, food intake and oral glucose tolerance were normalized in RYGB-treated Zucker fatty *fa/fa* rats to the levels of lean Zucker fatty *fa/+* controls, in association with increased glucagon-like peptide 1 (GLP-1) and insulin release. In contrast, while fatty liver was also normalized in RYGB-treated Zucker fatty *fa/fa* rats, their circulating levels of the liver enzyme alanine aminotransferase (ALT) remained elevated at the level of obese Zucker fatty *fa/fa* controls. These findings suggest that the leptin system is not required for the normalization of energy and glucose homeostasis associated with RYGB, but that its potential contribution to the improvements in liver health postoperatively merits further investigation.

## 1. Introduction

Bariatric surgery is currently the most effective treatment option against morbid obesity, with numerous prospective clinical studies showing that Roux-en-Y gastric bypass (RYGB) is associated with marked and sustained weight loss as well as long-term remission of type 2 diabetes and fatty liver disease [1,2,3,4,5]. Because RYGB reduces stomach size and excludes the duodenum from contact with ingested food, physical restriction and malabsorption of nutrients, respectively, were originally thought to mainly account for its beneficial effects on energy and glucose homeostasis [6]. With the aid of rodent models of RYGB, however, it is becoming increasingly evident that complex changes in various molecular, cellular, and systems processes take place postoperatively [7,8,9,10], better understanding of which may guide the development of more effective, noninvasive treatments against metabolic disease.

Leptin is a 16-kDa endocrine protein mainly released from white adipocytes and circulates in proportion to fat mass, thereby serving as a negative feedback signal to the brain about long-term energy stores [11,12]. Beyond its centrally-mediated effects on suppressing appetite and increasing energy expenditure [11,12], leptin also lowers blood glucose [13] and limits hepatic lipid accumulation [14,15]. Accordingly, leptin-deficient *ob/ob* mice have severe hyperphagic obesity, hyperglycemia, and fatty liver [16,17]. On the other hand, diet-induced obesity is thought to arise from the development of central leptin resistance as a result of persistently elevated circulating leptin levels [18,19] as well as from complex pro-inflammatory processes that directly interfere with hypothalamic leptin receptor signaling [20,21,22,23]. For these reasons, leptin supplementation to *ob/ob* mice normalizes their metabolic status [24,25,26,27], whereas leptin sensitizers such as neutralizing leptin antibodies [19], hypothalamic ER stress relievers [28,29,30], and other molecules [31,32] have taken center stage in obesity drug development. 

Because RYGB mainly reduces fat mass [33], it is associated with a marked reduction in circulating leptin levels [34,35,36]—even beyond chronic caloric restriction-induced weight loss alone [37,38,39,40,41,42]. Nevertheless, endogenous leptin action has been proposed to be enhanced postoperatively [43,44,45,46,47,48], thereby preventing the powerful counter-regulatory response to depletion of energy stores which normally leads to weight-regain [11,12]. Indeed, the disproportionately reduced circulating leptin levels associated with RYGB may in itself enhance endogenous leptin action by reversing central leptin resistance [46]. Further, the appetite suppressing effects of exogenous leptin are increased in RYGB-treated, diet-induced obese Wistar rats associated with an attenuation of hypothalamic inflammation and ER stress [45]. Studies using *ob/ob* mice directly aimed at assessing the requirement of the leptin system for the beneficial outcomes of RYGB on energy and glucose homeostasis, however, have yielded conflicting results [43,44]. Specifically, the sustained weight loss and food intake suppression following RYGB was found to be preserved in one study [44], but not in another [43], although in both studies RYGB failed to fully improve glycemic control [43,44]. In contrast, weight loss and enhanced insulin sensitivity [49] as well as improved fasting blood glucose levels and oral glucose tolerance [50] in leptin-unresponsive *db/db* mice [51], which lack the intracellular signaling domain unique to leptin b receptors due to an autosomal recessive point mutation in the leptin receptor gene [52], appear to be largely preserved following RYGB. 

Zucker fatty *fa/fa* rats are another genetic model of leptin receptor deficiency since they harbor an autosomal recessive point mutation in the leptin receptor gene—distinct from the *db/db* point mutation—which causes an inhibitory amino acid substitution in the extracellular domain common to all leptin receptor subtypes (a–f) [53,54,55]. As a result, Zucker fatty *fa/fa* rats are obese and hyperlipidemic [56,57] and exhibit markedly impaired oral glucose tolerance [57,58] as well as fatty liver [57,59]. Numerous studies have been performed aimed at assessing the metabolic effects of RYGB on Zucker fatty *fa/fa* rats [60,61,62,63,64] and on the inbred [65] Zucker diabetic fatty *fa/fa* rat strain [38,62,66,67,68,69,70,71,72,73,74,75,76,77,78,79,80,81], but their descriptions on food intake are generally either incomplete [60,61,62,63,64,66,67,68,71,78,79,81] or, in many studies, entirely missing [38,69,70,72,73,74,75,76,77,80]. Additionally, only a few of these studies incorporated a lean control group in the form of heterozygous Zucker fatty *fa/+* rats [60,62,63,76], which is essential if any conclusions are to be drawn about whether RYGB normalizes metabolic status. Surprisingly, all of these studies overlooked the role of leptin receptors in the lasting metabolic benefits associated with RYGB. We therefore directly asked if leptin receptors are required for RYGB to normalize energy and glucose homeostasis as well as fatty liver by using Zucker fatty *fa/fa* rats.

## 2. Materials and Methods 

### 2.1. Animals

**Figure 1:**
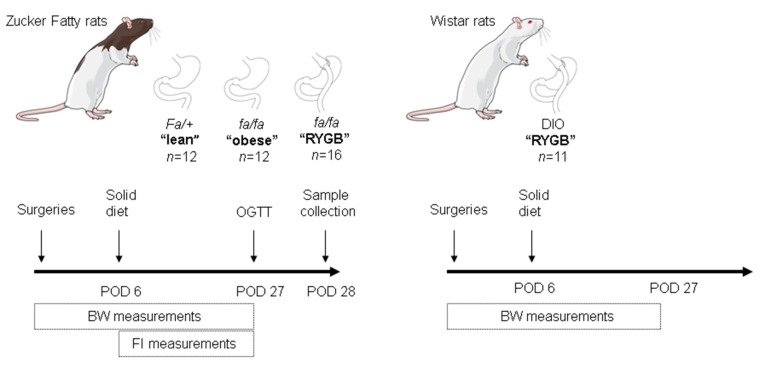
Schematic of experimental design. RYGB: Roux-en-Y gastric bypass; POD: postoperative day; BW: body weight; FI: food intake; OGTT: oral glucose tolerance test; DIO: diet-induced obese.

Twenty-eight male Zucker fatty *fa/fa* and 12 Zucker fatty *fa/+* rats were purchased from Charles River, France, aged 6 weeks. Data from part of these rats (16 Zucker fatty *fa/fa* and 5 Zucker fatty *fa/+* rats) were previously reported [62] and were incorporated into the present study to increase statistical power. All rats were individually housed under ambient humidity and a temperature of 22 °C in a 12 h light/dark cycle with free access to tap water and Purina 5008 Lab diet (Purina Mills, St. Louis, MO, USA, 16.7% of calories from fat), unless otherwise stated. An additional group of 11 previously phenotyped male Wistar that received RYGB at 11 weeks of age [82] were also incorporated into the present study (Figure 1). These rats were placed under identical housing and surgical conditions as Zucker fatty rats, but were given an Altromin C1090-60 high-fat diet (Altromin, Lage, Germany, 60% calories from fat) for 5-weeks preoperatively to induce obesity (478.8 ± 6.8 g) and a choice between an Altromin C1090-60 high-fat diet and an Altromin C1090-10 low-fat diet (Altromin, Lage, Germany, 10% kcal from fat) postoperatively to assess changes in food preference [82]. All experiments were reviewed and approved by the Animal Care Committee of the local government of Unterfranken, Bavaria, Germany (License numbers 55.2-2531.01-72/12 and 55.2-2532-2-467).

### 2.2. Surgeries

At 12 weeks of age, when Zucker fatty *fa/fa* rats developed obesity (443.1 ± 3.6 g), they were randomly allocated to RYGB (*n* = 16) or sham (*n* = 12) surgeries (Figure 1). The remaining 12 Zucker fatty *fa/+* rats were also scheduled for sham surgery (Figure 1). These group sizes are based on previous publications in which significant differences were observed [62,83]. All surgeries were performed under sterile conditions in 6 h fasted rats by an experienced bariatric surgeon after subcutaneous administration of 5 mg/kg carprofen as analgesia and intraperitoneal administration of 1.25 mg/kg amoxicillin as prophylactic antibiotic. Surgical anesthesia was induced with an isoflurane/oxygen mixture, and the abdomen was then opened using a midline laparotomy and closed following procedures using continuous suturing. 

For the sham procedure, the gastro-esophageal junction and small intestine were first mobilized. A gastrostomy on the anterior wall of the stomach was next performed followed by a jejunostomy as previously described [62,83]. For the RYGB procedure, the jejunum was first transected 15 cm below the pylorus. The stomach was next transected 3 mm below the gastro-esophageal junction, and the stomach remnant was subsequently closed to create the ∼15 cm biliopancreatic limb. To create the ∼80 cm alimentary limb, the aboral jejunum was anastomosed in an end-to-side fashion to the small stomach pouch (which was ∼5% of the original gastric volume). Finally, a 7-mm side-to-side jejuno-jejunostomy between the biliopancreatic limb and the alimentary limb at the level of the lower jejunum was performed to create a∼25 cm common channel as previously described [62,83]. 

### 2.3. Perioperative Care

Upon recovery from surgeries, rats were placed on a liquid diet (vanilla-flavored Ensure, Abbott Laboratories, IL, USA, 22% calories from fat) for 6 days postoperatively and then returned to their previous solid diet (Figure 1). For analgesia, they were subcutaneously administered 5 mg/kg carprofen once daily for the first 2 postoperative days. 

### 2.4. Metabolic Measurements

Food intake was measured daily from postoperative day 6, while body weight was measured daily throughout the 27-day monitoring period (Figure 1). An oral glucose tolerance test (OGTT) was performed at the beginning of the dark cycle on postoperative day 27 in Zucker fatty rats (Figure 1). For blood glucose measurements during the OGTT, a small tail vein incision was made in 8 h fasted rats, and a drop of blood was directly applied onto a glucometer (Breeze 2^®^ glucometer, Bayer, Zurich, Switzerland) at baseline and 15, 30, 60 and 120 min after 10 mL/kg body weight ingestion of a 25% glucose solution. A further 100 µL of tail vein blood was collected at each time-point into tubes containing EDTA. Plasma was isolated by centrifugation at 8000 rpm for 10 min at 4 °C and stored at −20 °C. Homeostatic model of insulin resistance (HOMA-IR) was calculated by the dividing the product of fasting plasma insulin (in µU/L) and blood glucose (in nmol/L) levels by 22.5 [84]. Matsuda–DeFronzo insulin sensitivity index (ISI-M) was calculated based on the results of the OGTT as follows:

ISI-M = 10,000/(*G*_0_ × *I*_0_ × *G*_mean_ × *I*_mean_)^1/2^ (1)

where *G* and *I* represents blood glucose (in mmol/dL) and plasma insulin (in mU/L) levels, respectively, and ‘0’ and ‘mean’ indicates fasting value and mean value during the OGTT, respectively [85]. 

### 2.5. Tissue Harvesting

At the 28th postoperative day, overnight-fasted Zucker Fatty rats were euthanized by isoflurane overdose 45 min after a fixed meal of 3 g Purina 5008 diet. Cardiac blood was collected into tubes containing EDTA and plasma was isolated by centrifugation at 8000 rpm for 10 min at 4 °C and stored at −20 °C. Epididymal white adipose tissue (eWAT) and retroperitoneal white adipose tissue (rWAT) were dissected according to a standardized protocol, weighed and summed to provide a measure of visceral WAT (vWAT) [86]. 

### 2.6. ELISAs

Plasma insulin was measured using an Ultrasensitive Rat Insulin ELISA kit (Mercodia AB, Uppsala, Sweden, #10-1251-10), plasma GLP-1 using a Rat GLP-1 ELISA kit (EMD Millipore, MA, USA, #EZGLP1T-36 K), plasma leptin using a Rat Leptin ELISA kit (Abcam, Cambridge, UK, #ab100773), plasma alanine transaminase (ALT) using a Rat ALT Simplestep^®^ ELISA kit (Abcam, Cambridge, UK #ab264579), and plasma aspartate transaminase (AST) using a Rat AST Simplestep^®^ ELISA kit (Abcam, Cambridge, UK, #ab263883) according to the manufacturer’s instructions.

### 2.7. Bomb Calorimetry

Feces were collected from Zucker fatty rats on postoperative day 28, dried in an oven and weighed. Fecal energy content (kcal/g) was then measured using ballistic bomb calorimetry.

### 2.8. Liver Histology

Freshly harvested liver from Zucker fatty rats was fixed with 4% paraformaldehyde for 24 h at room temperature and then embedded in paraffin blocks, cut into 2 μm-thick sections and mounted on glass slides for hematoxylin and eosin (H&E) staining according to a standard protocol. Representative images from each group were taken on a Keyence BZ-1000 microscope at a magnification of 20×. 

### 2.9. Statistics

Statistical analysis was performed using GraphPad PRISM Version 8^®^. Data are expressed as mean ± standard error of the mean (SEM). A one-way analysis of variance (ANOVA) with Sidak’s post hoc test or two-tailed, unpaired *t*-test was used to determine differences between groups. 

## 3. Results

### 3.1. Leptin Receptors Are Not Required for RYGB to Normalize Energy Homeostasis 

**Figure 2:**
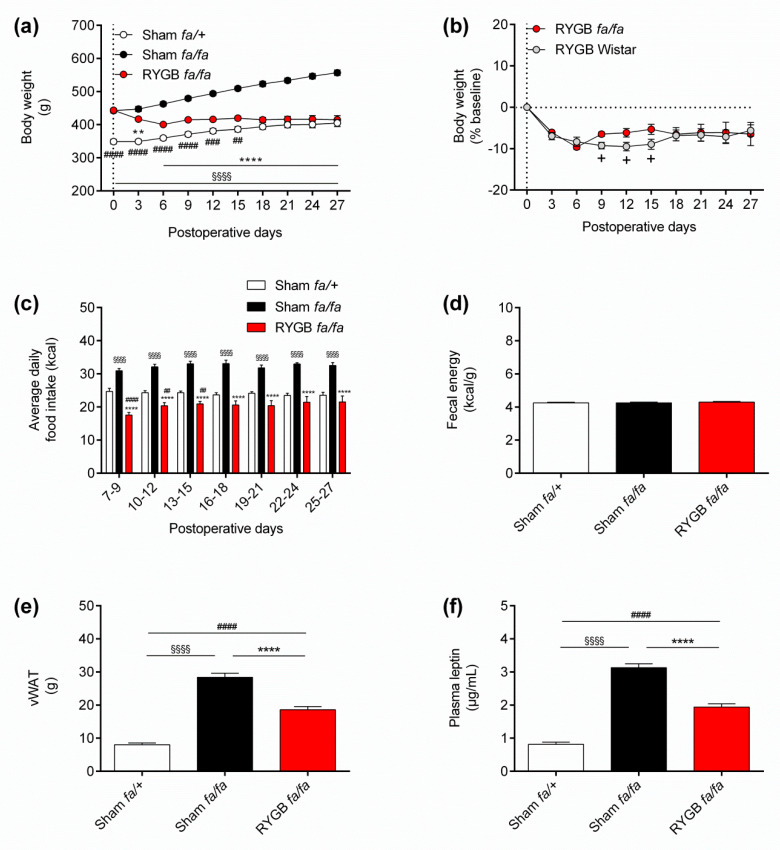
Leptin receptors are not required for RYGB to normalize energy homeostasis. (**a**) Body weight in grams (g) of sham-operated *fa/+* (lean) Zucker fatty rats (*n* = 12), sham-operated (obese) *fa/fa* Zucker fatty rats (*n* = 12), and RYGB-treated *fa/fa* Zucker fatty rats (*n* = 16) during the 27-day monitoring period. (**b**) Percentage (%) body weight change relative to baseline for RYGB-treated *fa/fa* rats (*n* = 16) in (**a**) and RYGB-treated Wistar rats (*n* = 11) from Dischinger et al. [82]. (**c**) Average daily food intake in kilocalories (kcal) in the rats from (**a**) during the 27-day monitoring period. (**d**) Fecal energy, (**e**) visceral white adipose tissue weight and (**f**) plasma leptin in the rats from (**a**) at postoperative day 28. Data are presented as mean ± SEM. Statistical significance was determined by one-way ANOVA with Sidak post hoc test in (**a**,**c**,**e**,**f**) and by two-tailed, unpaired *t*-test in (**b**). ^§§§§^ *p* < 0.0001 for sham-operated *fa/+* vs. sham-operated *fa/fa* Zucker fatty rats, ** *p* < 0.01 and **** *p* < 0.0001 for RYGB-treated *fa/fa* vs. sham-operated *fa/fa* Zucker fatty rats and ^####^ *p* < 0.0001, ^###^ *p* < 0.001 and ^##^ *p* < 0.01 for RYGB-treated *fa/fa* vs. sham-operated *fa/+* Zucker fatty rats, and + *p* < 0.05 for RYGB-treated *fa/fa* Zucker fatty vs. RYGB-treated Wistar rats.

We first assessed body weight trajectories in lean, obese, and RYGB-treated Zucker fatty rats over the course of a 27-day monitoring period (Figure 1). Consistent with the leptin receptor-deficient state of Zucker fatty *fa/fa* rats, baseline body weights of RYGB-treated (443.7 ± 2.8 g) and obese (442.3 ± 6.7 g) rats were significantly higher than lean rats (348.8 ± 8.1 g; *p* < 0.0001 for both comparisons) (Figure 2a). From postoperative day 3 onwards, both obese and lean rats progressively gained body weight (Figure 2a). In contrast, RYGB-treated rats lost body weight until postoperative day 6, which then largely stabilized and eventually converged with lean rats by study close at postoperative day 27 (414.7 ± 12.5 g vs. 405.0 ± 10.7 g, respectively; *p* = 0.99) (Figure 2a).

To ascertain if the effect on body weight of our RYGB rat model is similar when leptin receptors are intact, we incorporated data previously obtained from RYGB-treated, diet-induced obese Wistar rats [82]. Because these rats weighed significantly more than RYGB-treated Zucker fatty *fa/fa* rats at baseline (*p* < 0.01), body weights for this comparison were expressed as percentage change (Figure 2b). This revealed similar percentage weight loss for both groups during postoperative days 0–6, but RYGB-treated Zucker fatty *fa/fa* rats then slightly regained body weight at postoperative day 9, whereas RYGB-treated Wistar rats did so later at postoperative day 18 (Figure 2b). As a result, percentage weight loss was significantly greater for RYGB-treated Wistar rats during postoperative days 9–15 (*p* < 0.05) (Figure 2b). However, percentage weight loss between groups was similar from postoperative days 18–27, such that RYGB-treated Wistar rats weighed 6.7 ± 2.8% less, whereas RYGB-treated Zucker fatty *fa/fa* rats weighed 5.6 ± 1.4% less compared with baseline by study close at postoperative day 27 (*p* = 0.78) (Figure 2b).

In accordance with the changes in body weights between Zucker fatty rat groups, average daily food intake of RYGB-treated rats was similar to lean rats (i.e., normalized) from postoperative days 16–18 onwards, whereas it was always significantly lower than obese rats (*p* < 0.0001) up until study close at postoperative days 25–27 (21.5 ± 1.8 kcal vs. 32.5 ± 0.9 kcal per day, respectively; *p* < 0.0001) (Figure 2c). Notably, average daily food intake of RYGB-treated rats was significantly lower than lean rats from postoperative days 7–9 (*p* < 0.0001) to postoperative days 13–15 (*p* < 0.01), which we attribute to the longer time it requires for the reconfigured gastrointestinal tract to fully heal. 

To exclude malabsorption as a cause of changes in body weight in our RYGB rat model, we measured energy content in fecal samples collected from Zucker fatty rats at postoperative day 28 by bomb calorimetry. This revealed negligible differences between groups (Figure 2d). 

To determine the effects of RYGB on adiposity, vWAT was dissected and weighed from Zucker fatty rats following euthanasia at postoperative day 28. This revealed that vWAT of obese rats weighed significantly more than lean and RYGB-treated rats (28.4 ± 1.2 g vs. 8.0 ± 0.6 g and 18.6 ± 1.0 g, respectively; *p* < 0.0001 for both comparisons) (Figure 2e). Sidak post hoc test also revealed that the vWAT of RYGB-treated rats weighed significantly more than lean rats (*p* < 0.0001) (Figure 2e). These group differences in vWAT weights were fully reflected in plasma leptin levels, which were the highest for obese rats (3.1 ± 0.11 µg/mL), followed by RYGB-treated rats (1.9 ± 0.10 µg/mL), and then by lean rats (0.8 ± 0.07 µg/mL) (Figure 2f).

### 3.2. Leptin Receptors Are Not Required for RYGB to Normalize Oral Glucose Tolerance 

**Figure 3:**
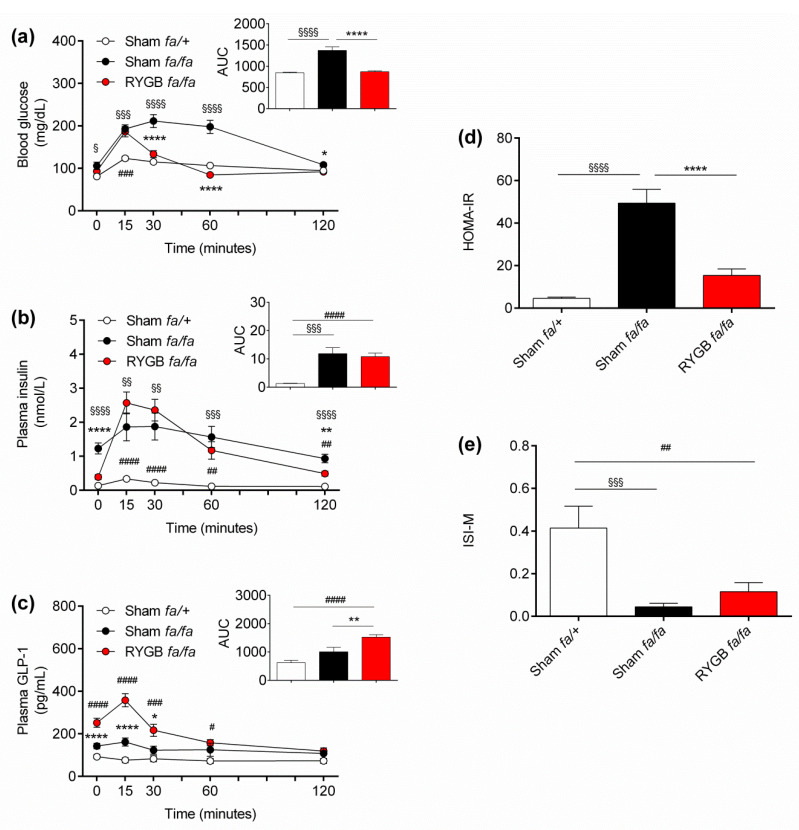
Leptin receptors are not required for RYGB to normalize oral glucose tolerance. (**a**) Blood glucose and the associated area under the curve (AUC), (**b**) plasma insulin and the associated AUC and (**c**) plasma GLP-1 and the associated AUC during an oral glucose tolerance test in sham-operated (lean) *fa/+* Zucker fatty rats (*n* = 12), sham-operated (obese) *fa/fa* Zucker fatty rats (*n* = 12), and RYGB-treated *fa/fa* Zucker fatty rats (*n* = 16) at postoperative day 27. (**d**) HOMA-IR and (**e**) ISI-M indices calculated from the rats in (**a**). Data are presented as mean ± SEM. Statistical significance was determined by one-way ANOVA with Sidak post hoc test. ^§§§§^ *p* < 0.0001, ^§§§^ *p* < 0.001, ^§§^ *p* < 0.01 and ^§^ *p* < 0.05 for sham-operated *fa/+* vs. sham-operated *fa/fa* Zucker fatty rats, **** *p* < 0.0001, ** *p* < 0.001 and * *p* < 0.05 for RYGB-treated *fa/fa* vs. sham-operated *fa/fa* Zucker fatty rats and ^####^ *p* < 0.0001, ^###^ *p* < 0.001, ^##^ *p* < 0.01, ^#^ *p* < 0.05 for RYGB-treated fa/fa vs. sham-operated *fa/+* Zucker fatty rats.

Next, to determine if leptin receptors are required for RYGB to normalize glycemic control, an OGTT was performed at postoperative day 27 (Figure 1). Fasting blood glucose levels at baseline were significantly higher for obese compared with lean rats (105.8 ± 8.6 mg/dL vs. 80.8 ± 3.3 mg/dL, respectively; *p* = 0.01), whereas they were similar for RYGB-treated rats (92.9 ± 3.7 mg/dL) compared with both lean (*p* = 0.23) and obese (*p* = 0.27) rats (Figure 3a). During the OGTT, blood glucose levels peaked for lean rats at 15 min and then steadily declined by 120 min (Figure 3a). In contrast, blood glucose levels peaked for obese rats at 30 min but remained elevated at 60 min before slowly declining by 120 min (Figure 3a). The blood glucose excursion curve for RYGB-treated rats during the OGTT was markedly different to both lean and obese rats peaking at 15 min, then steeply dropping to below baseline values at 60 min and then returning to near baseline values at 120 min (Figure 3a). This blood glucose dynamic is similar to that described for RYGB-treated, non-diabetic/diabetic patients during a mixed meal tolerance test and has been attributed to increased glucose absorption and clearance [87,88]. Further, area under the curve (AUC) analysis revealed a normalization of integrated blood glucose levels during the OGTT for RYGB-treated rats (Figure 3a).

Concerning plasma insulin levels, obese rats were hyperinsulinemic at baseline (1.2 ± 0.2 nmol/L), with significantly higher plasma insulin levels compared with both lean (0.14 ± 0.01 nmol/L) and RYGB-treated (0.39 ± 0.07 nmol/L) rats (*p* < 0.0001 for both comparisons) (Figure 3b). During the OGTT, plasma insulin levels only slightly rose for lean rats peaking at 15 min and then gradually declined by 120 min (Figure 3b). For obese rats, plasma insulin levels peaked at 30 min, but remained elevated at 60 min before gradually declining by 120 min (Figure 3b). Again, the plasma insulin curve for RYGB-treated rats during the OGTT was qualitatively different from both lean and obese rats with plasma insulin levels peaking at 15 min, but remaining elevated at 30 min, before steeply declining to near baseline levels by 120 min (Figure 3b). This could be explained by the increased release of the incretin GLP-1 in RYGB-treated rats, which also peaked 15 min into the OGTT (Figure 3c). Again, the plasma profile of insulin and GLP-1 during the OGTT in RYGB-treated rats is similar to that described for RYGB-treated, non-diabetic/diabetic patients during a mixed-meal tolerance test [87,88]. AUC analysis revealed similar integrated circulating insulin levels during the OGTT for RYGB-treated compared with obese rats, which were significantly higher than lean rats (*p* < 0.0001 for both comparisons) (Figure 3b). On the other hand, AUC analysis revealed the highest integrated circulating GLP-1 levels during the OGTT for RYGB-treated rats compared with both lean (*p* < 0.0001) and obese (*p* < 0.001) rats (Figure 3c). 

Based on the OGTT data, we calculated HOMA-IR indices, as an indicator of insulin resistance [84], and found them to be normalized in RYGB-treated rats (Figure 3d). In contrast, ISI-M indices, as an indicator of insulin sensitivity [85], were significantly higher for lean rats compared with both obese and RYGB-treated rats (*p* < 0.0001 for both comparisons) (Figure 3e). 

### 3.3. Leptin Receptors Might Be Required for RYGB to Normalize Liver Health 

**Figure 4:**
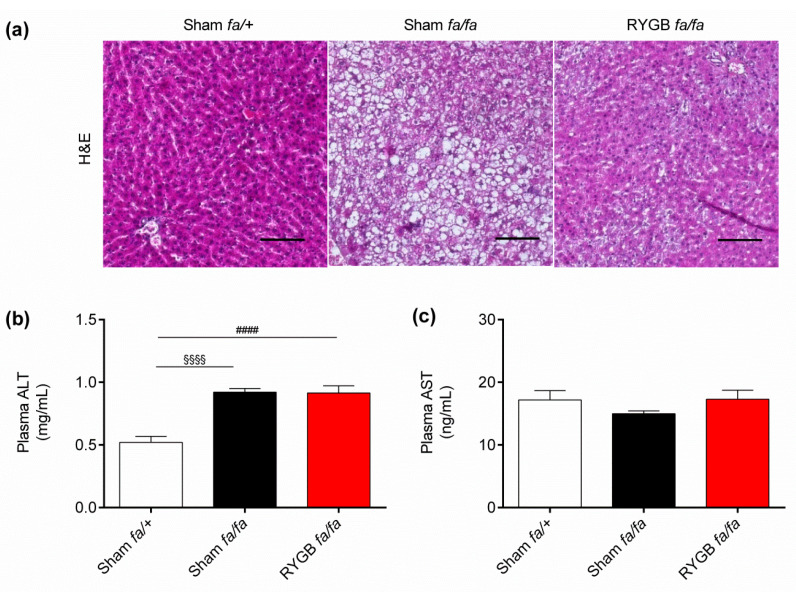
Leptin receptors might be required for RYGB to normalize liver health. (**a**) Representative hematoxylin and eosin (H&E) staining of liver sections, (**b**) plasma alanine transferase (ALT), and (**c**) plasma aspartate transferase (AST) of sham-operated *fa/+* (lean) Zucker fatty rats (*n* = 12), sham-operated (obese) *fa/fa* Zucker fatty rats (*n* = 12), and RYGB-treated *fa/fa* Zucker fatty rats (*n* = 16) at postoperative day 28. Data are presented as mean ± SEM. Scale bar: 50 µm. Statistical significance was determined by one-way ANOVA with Sidak post hoc test. ^§§§§^ *p* < 0.0001 for sham-operated *fa/+* vs. sham-operated *fa/fa* Zucker fatty rats and ^####^ *p* < 0.0001 for RYGB-treated *fa/fa* vs. sham-operated *fa/+* Zucker fatty rats.

Finally, to determine if leptin receptors are required for RYGB to normalize liver health, we performed H&E staining on paraffin-embedded liver sections and also measured circulating levels of the liver enzymes ALT and AST as indicators of liver damage. This revealed that obese rats had markedly more hepatic lipid deposits than lean rats (Figure 4a), in line with previous studies [57,59], whereas RYGB-treated rats had similar hepatic lipid deposits compared with lean rats (Figure 4a). Interestingly, although circulating levels of ALT were higher in obese rats compared with lean rats (0.92 ± 0.03 mg/mL vs. 0.52 ± 0.05 mg/mL; *p* < 0.0001), again in line with previous studies [57,59], they remained elevated in RYGB-treated rats (0.91 ± 0.06 mg/mL) (Figure 4b). In contrast, circulating levels of AST were similar between groups (Figure 4c). 

## 4. Discussion

Zucker fatty *fa/fa* rats harbor an autosomal recessive point mutation in the leptin receptor gene that negatively affects the extracellular domain common to all leptin receptor subtypes (a–f) [53,54,55], making them an established genetic model of leptin receptor deficiency. We obtained evidence using these rats that the leptin system is not required for RYGB to normalize energy and glucose homeostasis, whereas it might play an independent role in improving liver health. 

The first studies aimed at assessing the requirement of the leptin system in the improvements in energy and glucose homeostasis associated with RYGB used leptin-deficient *ob/ob* mice [43,44]. Our findings do not align with those of Hao et al. [43] who showed that the changes in body weight of *ob/ob* and diet-induced obese mice following RYGB were markedly different. A potential reason for the discrepancy with our findings is that the RYGB mouse model of Hao et al. [43] is not associated with suppression of food intake and instead causes malabsorption, unlike the RYGB-treated Zucker fatty *fa/fa* rats described here. Our findings do, however, agree with those of Mokadem et al. [44] who showed that RYGB induced sustained weight loss in *ob/ob* mice up until the end of the 6-week monitoring period and reduced average food intake by 23%. In contrast, body weight and oral glucose tolerance in the RYGB-treated *ob/ob* mice of Mokadem et al. [44] were not normalized. The reasons for the discrepancies with our findings could be due to species differences or the degree of diminished leptin action between *ob/ob* mouse (absolute) and Zucker fatty *fa/fa* rat (severely diminished) models. In this regard, while Zucker fatty *fa/fa* rats reduce food intake upon central leptin administration at pharmacological doses [89,90], they fail to do so to peripherally administered leptin [90]. Therefore, it is unlikely that any residual circulating leptin action could contribute to the suppression of food intake in RYGB-treated Zucker fatty *fa/fa* rats. 

Because RYGB is very technically demanding to execute in mice, more studies have been performed aimed at assessing its metabolic effects in Zucker fatty *fa/fa* [60,61,62,63,64] and Zucker diabetic fatty *fa/fa* [38,62,66,67,68,69,70,71,72,73,74,75,76,77,78,79,80] rats. These studies, however, like those in *db/db* mice [49,50], were not directly aimed at assessing the requirement of the leptin system in the improvements in energy and glucose homeostasis associated with RYGB. This is most likely why their details of food intake are either generally incomplete [60,61,62,63,64,66,67,68,71,78,79,81], or missing [38,49,50,69,70,72,73,74,75,76,77,80], and their study conclusions are unrelated to leptin. Our findings differ from the studies showing a lack of sustained weight loss associated with RYGB [38,64,68,71,74,75,78,80], but are in line with the majority that do [61,62,63,66,67,69,72,76,79,81]. Further, our findings are consistent with the studies showing suppression of average food intake associated with RYGB over the postoperative monitoring period [62,66,67,71,78,79,81]. The reasons for the discrepancies between studies could be due to the age of rats when surgeries were performed, the postoperative maintenance diet, the duration of the postoperative monitoring period, as well as the RYGB model, which can vary significantly between laboratories [91]. Nevertheless, our study extends previous work by showing a clear normalization of food intake by RYGB that is sustained at a late postoperative time-point when body weight is also normalized by the procedure. 

Importantly, we could demonstrate that the changes in body weight over the course of a 27-day monitoring period in RYGB-treated Zucker fatty *fa/fa* rats largely overlapped with diet-induced obese Wistar rats that have intact leptin receptors. This is in line with other studies showing similar 13% weight loss 11 days after RYGB in Zucker diabetic fatty *fa/fa* rats and Sprague-Dawley rats [67], as well as similar body weight trajectories over a lengthier 4-week postoperative monitoring period in these rats [68,78]. In contrast, the potent weight lowering and appetite suppressing effects of various small molecule leptin sensitizers in diet-induced obese mice are lost in both *ob/ob* and *db/db* mice [28,29,32]. These findings collectively argue against sensitization to endogenous leptin playing a causal role in the outcome of RYGB on energy homeostasis. A recent study using diet-induced obese Wistar rats, however, reached the opposite conclusion [45]. A major limitation of this study is that it focused only on the effects of exogenous leptin treatment [45]. Moreover, food intake in the RYGB-treated rats of Chen et al. [45] returned to the levels of sham-operated counterparts and was not affected when central leptin resistance was induced pharmacologically [45].

With regards to glucose homeostasis, previous studies have consistently shown lower fasting blood glucose and/or plasma insulin levels in RYGB-treated Zucker fatty *fa/fa* and Zucker diabetic fatty *fa/fa* rats compared with sham-operated counterparts [38,61,63,64,66,67,68,69,71,72,76,79,81]. Additionally, studies with a longitudinal design have shown that RYGB improves oral glucose tolerance at postoperative days 21 and 30 compared with baseline [74,79]. Because we included a lean control group in the form of sham-operated *fa/+* Zucker fatty rats, we could show a normalization of fasting plasma insulin levels and HOMA-IR indices associated with RYGB, which is consistent with a previous study [63]. We further found normalized oral glucose tolerance, which differs from a previous study in Zucker diabetic fatty *fa/fa* rats [76]. This discrepancy can possibly be attributed to strain differences, since Zucker diabetic fatty *fa/fa* rats additionally harbor a point mutation that affects insulin transcription in pancreatic beta cells [92], rendering them genuinely diabetic unlike Zucker fatty *fa/fa* rats [65]. In contrast, we did not find significantly higher ISI values in RYGB-treated Zucker *fa/fa* rats compared with sham-operated counterparts, which differs from a previous study in which it was higher [61]. While we cannot offer an explanation for this discrepancy, the evidence from studies on Zucker fatty and Zucker diabetic fatty *fa/fa* rats generally suggests that leptin receptors are not required for the improvement/normalization of glucose homeostasis associated with RYGB [38,61,63,64,66,67,68,69,71,72,74,76,79,81]. 

The global prevalence of fatty liver disease has risen dramatically alongside that of obesity, and there currently exists no specific medical treatment [93]. Numerous prospective clinical studies have shown, however, that RYGB is associated with a marked improvement in liver health as determined histologically and reflected by a normalization of circulating ALT and AST levels [94]. Similarly, there is ample evidence that leptin protects against accumulation of liver fat [95], with rodent studies implicating leptin receptors in the hypothalamus [15] and the dorsal vagal complex [14]. Further, leptin sensitizers markedly improve fatty liver and lower circulating ALT/AST levels in diet-induced obese mice [19,28,29]. We, however, found lower liver fat in RYGB-treated Zucker fatty *fa/fa* rats compared with sham-operated counterparts, similar to a previous study [64] and also to RYGB-treated *ob/ob* mice [44], suggesting that the leptin system is not required for this metabolic benefit postoperatively. In contrast, circulating ALT levels between RYGB-treated and sham-operated Zucker fatty *fa/fa* rats were similar. While this might suggest that leptin receptors are required for the complete normalization of liver health associated with RYGB, a previous study documented lower circulating ALT levels in RYGB-treated Zucker diabetic fatty *fa/fa* rats compared with sham-operated counterparts [71]. Clearly, more in-depth preclinical studies are needed on the extent to which RYGB improves fatty liver disease, including its impact on inflammation and fibrosis, and the potential role played by the leptin system. 

If leptin is not required for the improved energy and glucose homeostasis associated with RYGB, then this raises the obvious question as to which peripheral factors are required. We confirmed that circulating levels of the anorexigenic and incretin gut hormone GLP-1 are increased by RYGB in Zucker fatty/Zucker diabetic fatty *fa/fa* rats [61,62,68,75,78,79]. However, rodent studies have shown that like leptin receptors, GLP-1 receptors are not required for the effects of RYGB on body weight and glycemic control [96,97], although there is pharmacological evidence suggesting that GLP-1 receptor signaling is required for the postoperative improvement in oral glucose tolerance [98]. Similarly, the farnesoid X receptor (FXR), which is a target of bile acids, is required for the improvements in glycemic control associated with RYGB in mice [99]. Still, the peripheral factors required for the improvements in energy homeostasis associated with RYGB remain unknown, and their identification represents an important future line of investigation. Notably, Zucker fatty *fa/fa* or Zucker diabetic fatty *fa/fa* rats, with their sustained suppression of food intake following RYGB [66], may be the ideal model for such investigations. This is because food intake suppression in RYGB-treated, diet-induced obese mice and rats tends to diminish over time [9,45,67,82,97,99,100] or is even absent [43,78,96].

Strengths of the present study include the well-powered group sizes allowing for robust statistical comparisons to be performed, as well as the incorporation of a lean control group. Another study strength is the detailed reporting of body weight and food intake absent in previous studies with RYGB-treated Zucker fatty *fa/fa* or Zucker diabetic fatty *fa/fa* rats. We also directly compared body weight changes following RYGB in leptin receptor-deficient and replete states, revealing the non-essential role of the leptin system in the normalization of energy homeostasis postoperatively. A limitation of the present study is that despite achieving the degree of food intake suppression associated with RYGB in patients [101], the 30–40% weight loss characteristic of the procedure [3] was not reached. However, when factoring in the rapid weight gain of sham-operated Zucker fatty *fa/fa* rats, RYGB-treated rats weighed 25.5 ± 2.2% less, which resembles the clinical outcome. Additionally, we did not directly compare the effects of RYGB on glucose homeostasis and fatty liver with a diet-induced obese group in which endogenous leptin action could be restored or enhanced, which needs to be performed in future studies. Finally, we did not challenge RYGB-treated Zucker fatty *fa/fa* rats with a high-fat diet postoperatively, which could have uncovered a more prominent role for the leptin system in preventing weight regain [44].

In summary, we have presented evidence arguing against the requirement of the leptin system in the normalization of energy and glucose homeostasis associated with RYGB, which is consistent with the majority of previous studies in Zucker fatty *fa/fa* and Zucker diabetic fatty *fa/fa* rats as models of leptin receptor deficiency [61,62,63,66,67,69,72,76,79,81] and thus places them in a new light. 

## Data Availability

The data in this manuscript are available on request from the corresponding authors.
